# A mixed-methods evaluation of a patient navigation intervention to support lung cancer prevention in LGBTQ+ adults

**DOI:** 10.1093/ntr/ntag136

**Published:** 2026-06-19

**Authors:** Nicholas R Murphy, Brandon Omernik, Madison Snidarich, Jaimee L Heffner, Elle Brooks, Kristina Crothers, Meagan C Brown, Matthew Triplette

**Affiliations:** Public Health Sciences Division, Fred Hutchinson Cancer Center, Seattle, WA 98109, United States; Division of Pulmonary, Critical Care and Sleep Medicine, University of Washington, Seattle, WA 98195, United States; Division of Pulmonary, Critical Care, Allergy and Sleep Medicine, University of California, San Francisco, San Francisco, CA 94143, United States; Living Tobacco Free Services, Fred Hutchinson Cancer Center, Seattle, WA 98109, United States; Public Health Sciences Division, Fred Hutchinson Cancer Center, Seattle, WA 98109, United States; Public Health Sciences Division, Fred Hutchinson Cancer Center, Seattle, WA 98109, United States; Seattle LGBTQ+ Center, Seattle, WA 98122, United States; Division of Pulmonary, Critical Care and Sleep Medicine, University of Washington, Seattle, WA 98195, United States; Kaiser Permanente Washington Research Institute, Seattle, WA 98101, United States; Public Health Sciences Division, Fred Hutchinson Cancer Center, Seattle, WA 98109, United States; Division of Pulmonary, Critical Care and Sleep Medicine, University of Washington, Seattle, WA 98195, United States; Living Tobacco Free Services, Fred Hutchinson Cancer Center, Seattle, WA 98109, United States

**Keywords:** cessation, lung cancer, cancer screening, health disparities, qualitative research

## Abstract

**Introduction:**

The lesbian, gay, bisexual, transgender, and queer/questioning (LGBTQ+) community represents a high-risk group for lung cancer given higher smoking rates. We evaluated implementation of a pilot patient navigation intervention for smoking cessation services combined with lung cancer screening (LCS) care for eligible LGBTQ+ adults.

**Methods:**

We completed a concurrent mixed–methods evaluation of the intervention to enhance adaptation and future scale. Forty-one LGBTQ+-identifying individuals who were eligible for LCS and currently smoking were enrolled, completed baseline surveys, and underwent the intervention for a 90-day period. Thirty-four participants completed post-intervention surveys and 15 were invited for semi-structured interviews. We conducted a thematic analysis of qualitative data, integrated with quantitative and qualitative survey data through joint displays, guided by the Health Equity Implementation Framework.

**Results:**

We identified 14 themes in three topical domains. Key themes related to *Intervention Success* included: flexible communication; iterative contact through a longitudinal relationship; improved confidence and comfort; multimodal and tailored elements. Themes related specifically to *LGBTQ+ Care* included: LGBTQ+-specific social challenges; the impact of historic discrimination; emphasis on non-judgmental care; and varied responses to LGBTQ+-specific tailoring. Themes related to *Future Adaptations* included: more education and information on the LGBTQ+-tailored approach; synergy with trusted primary care clinicians; upfront information on financial risks; community connection and peer support; and variable need for more intense support.

**Conclusions:**

LGBTQ+-tailored patient navigation is an acceptable and well-suited intervention to facilitate and integrate LCS care and smoking cessation. Further adaptation should include more education and incorporate components of community support.

## Introduction

Lung cancer remains the leading cause of cancer-related death in the United States.^1^ As more than 80% of all lung cancer incidence in the United States is related to cigarette use,^2^^,^^3^ primary prevention through smoking cessation is the most effective tool to reduce lung cancer mortality. Evidence also supports lung cancer screening (LCS) as secondary prevention and screening with annual low-dose chest CT can reduce lung cancer mortality by 20% in a high-risk population.^4^^,^^5^ LCS and smoking cessation have synergistic contributions to lung cancer risk reduction.^6^ Despite overall declines in both lung cancer incidence and mortality over the past three decades paralleling population-level reductions in smoking, both smoking and lung cancer disparities have persisted.

Overall, there is higher historic and current tobacco use in people who identify as lesbian, gay, bisexual, transgender, queer/questioning (LGBTQ+), thus community members are more likely to be eligible for LCS and benefit from smoking cessation assistance.^7–9^ Contributing factors to increased smoking likely include intersecting social determinants, minority stress, community and social influences, and targeted marketing from tobacco companies.^10–15^ Studies have revealed other disparities along the lung cancer care continuum in LGBTQ+ people, such as delays in diagnosis, which raise the imperative of prevention and early detection.^16–18^

After defining community needs and priorities from diverse LGBTQ+ stakeholders within the King County, Washington area, we co-created an intervention to support lung cancer prevention, incorporating both smoking cessation and LCS assistance through a patient navigation model.^19^ Supportive care was delivered longitudinally by a navigator who was an experienced tobacco treatment specialist. The navigation intervention was delivered over at least a 3-month period. As primary outcomes, we demonstrated that 3 months of navigation improved up-to-date LCS completion from 22% to 55%, led to significant reductions in smoking and *≥*30 day biochemically verified smoking cessation in 18%, and was highly acceptable.^20^ In this study, we present the mixed-methods implementation evaluation used to inform adaption and refinement prior to scale.

## Methods

### Study design

This is a mixed-methods evaluation of intervention implementation integrating survey and interview data from pilot trial participants. Eligible participants were individuals who self-identified as LGBTQ+; primarily resided in King County, Washington; reported current cigarette smoking (any cigarette smoking within the last 30 days at enrollment); and met additional US Preventive Services Task Force 2021 criteria for LCS: age 50–80 years, and a self–reported tobacco smoking history equivalent to at least 20 pack-years.^21^ Participants were enrolled between June 2023 and July 2024. This study was conducted within the University of Washington/Fred Hutchinson Cancer Center (UW/FHCC) multisite LCS program, which serves several Seattle–area affiliated hospitals and clinics.^22^^,^^23^ Participants were recruited with the assistance of community-based organizations serving the LGBTQ+ population of King County. All participants were recruited from community settings and were not prior participants in the UW/FHCC LCS program. Further details of the study design and recruitment have been previously described.^20^ All participants provided written informed consent, and the study was approved by Fred Hutchinson Cancer Center Institutional Review Board (IR 10815) and registered at ClinicalTrials.gov prior to participant enrollment (NCT05304390). All participants were invited to complete pre- and post-intervention surveys and 15 were purposively sampled to complete post-intervention interviews. The qualitative components of the study are reported according to Consolidated Criteria for Reporting Qualitative Research guidance.^24^

### Intervention: Patient navigation

The navigation intervention is described in a prior manuscript.^20^ Briefly, the intervention was co-designed with community partners and LGBTQ+ community members to support LCS completion and offer longitudinal smoking cessation. Broadly, it included domains of patient education; ongoing reminders and touchpoints regarding LCS activities; longitudinal smoking cessation support consistent with National Comprehensive Cancer Network guidelines and inclusive of behavioral counseling, nicotine replacement, linkage to prescription medication; and individualized barrier assessment and barrier-directed support.^25^ The intervention was formalized in a handbook and delivered by a single navigator who is also a certified tobacco treatment specialist who met with participants on the day of enrollment and communicated with them thereafter by secure patient portal messaging, telehealth or telephone based on program-defined touchpoints and patient preferences. Participants were requested to return for an in-person visit at day 90. Fidelity of patient navigation was measured through a 9-item checklist with planned observation of approximately 20% of initial visits, and EHR-based review of navigation activities to ensure they aligned with handbook defined touch-points.

### Surveys and interviews

Participants completed a pre-intervention survey at enrollment that included demographics, questions on health history, preferences for health information, and interactions with providers. Sex assigned at birth and gender identity were collected via National Academies of Sciences, Engineering, and Medicine standards.^26^ Further granularity on sexual orientation and gender identity was collected via a response item that allowed multiple selections and free-text responses. At day 90, participants completed a post-intervention survey that included updated smoking behavior and used validated measures of overall intervention acceptability,^27^ and satisfaction with the interpersonal relationship with the navigator,^28^ to assess the intervention implementation. Both pre- and post-intervention surveys included two validated measures of LCS knowledge for comparison, the 12-item LCS-12 and the 7-item LCS knowledge score (LKS).^29^ The post-survey also included questions to elicit feedback on the intervention and interactions with the navigator and two free-text open-ended questions eliciting what the participants liked most and least about the intervention.

Interviews were conducted with 15 enrolled participants to ensure thematic saturation. Participants who indicated willingness to participate in interviews on post-test surveys (31 of 34) were reviewed and purposively sampled to ensure diversity across LGBTQ+ identities, gender, and race/ethnicity. Purposive sampling was chosen rather than a random sampling strategy to ensure diverse viewpoints were included. Interviews were conducted over Zoom by a research coordinator and experienced interviewer (M.S.) with no prior relationship with participants using a semi-structured interview guide created by the study team through consensus, based on the Health Equity Implementation Framework to cover key determinants of a healthcare intervention’s implementation.^30^ The framework integrates an intervention explanatory framework (Integrated-Promiting Action of Research Implementation in Health Services) with a socioecologic framework considering disparities at multiple levels. Interviews lasted ~1 hour and were audio recorded and professionally transcribed. Transcripts were reviewed by qualitative team for accuracy and corrections but not returned to participants.

### Survey and interview analysis

Participant data were managed through a password-protected REDCap electronic data capture tool. Survey data are reported in summary measures as percentages or medians with interquartile ranges. Transcripts were analyzed using Atlas. Ti version 9 software (ATLAS.ti Scientific Software Development GmbH) using principles of thematic analysis.

Three authors (N.R.M., B.O., M.T.) formed the analysis team using a mixed deductive and inductive approach. The analysis team created a preliminary set of codes informed by the interview prompts and guided by the Health Equity Implementation Framework domains of “Innovation Factors,” “Clinical Encounter,” “Patient Factors,” and “Context Factors.” N.R.M. and B.O. coded four initial transcripts with these preliminary codes and met to modify and expand the codebook. This set of updated codes was reviewed with the analysis team as the final consensus codebook. All transcripts were double-coded with N.R.M. and B.O. coding each transcript independently with the consensus codebook further iterationated as needed. The analysis team met after completion of double-coding of all transcripts by N.R.M. and B.O. with resolution of any remaining discrepancies through consensus. Codes were then organized into a matrix which was analyzed using constant comparison to reduce the data to memos. As a final analytic step, relevant quantitative data from the pre- and post-surveys as well as free-text responses from the post-survey were integrated into the matrix in a joint display that allowed triangulation with the memos to inform final themes and larger topical groupings.

## Results

Forty-one participants enrolled and completed the baseline survey and 34 (83%) returned to complete the post-intervention survey at day 90. Fifteen participants were invited and completed interviews occurring between 16 and 24 weeks after baseline (Figure 1). There was high intervention fidelity among nine observed participant visits: six had 9/9 elements completed and three had 8/9 elements completed; 40/41 participants had appropriate EHR documentation per navigator handbook. The median age of participants was 60, the majority (76%) were assigned male at birth, and there was broad representation across LGBTQ+ identities. Participants in post-surveys and interviews appeared similar to the overall cohort across demographic characteristics (Table 1). Fourteen themes emerged from analysis in three groups: Implementation Success, LGBTQ+ Care, and Future Adjustments (Table 2).

**Figure 1:**
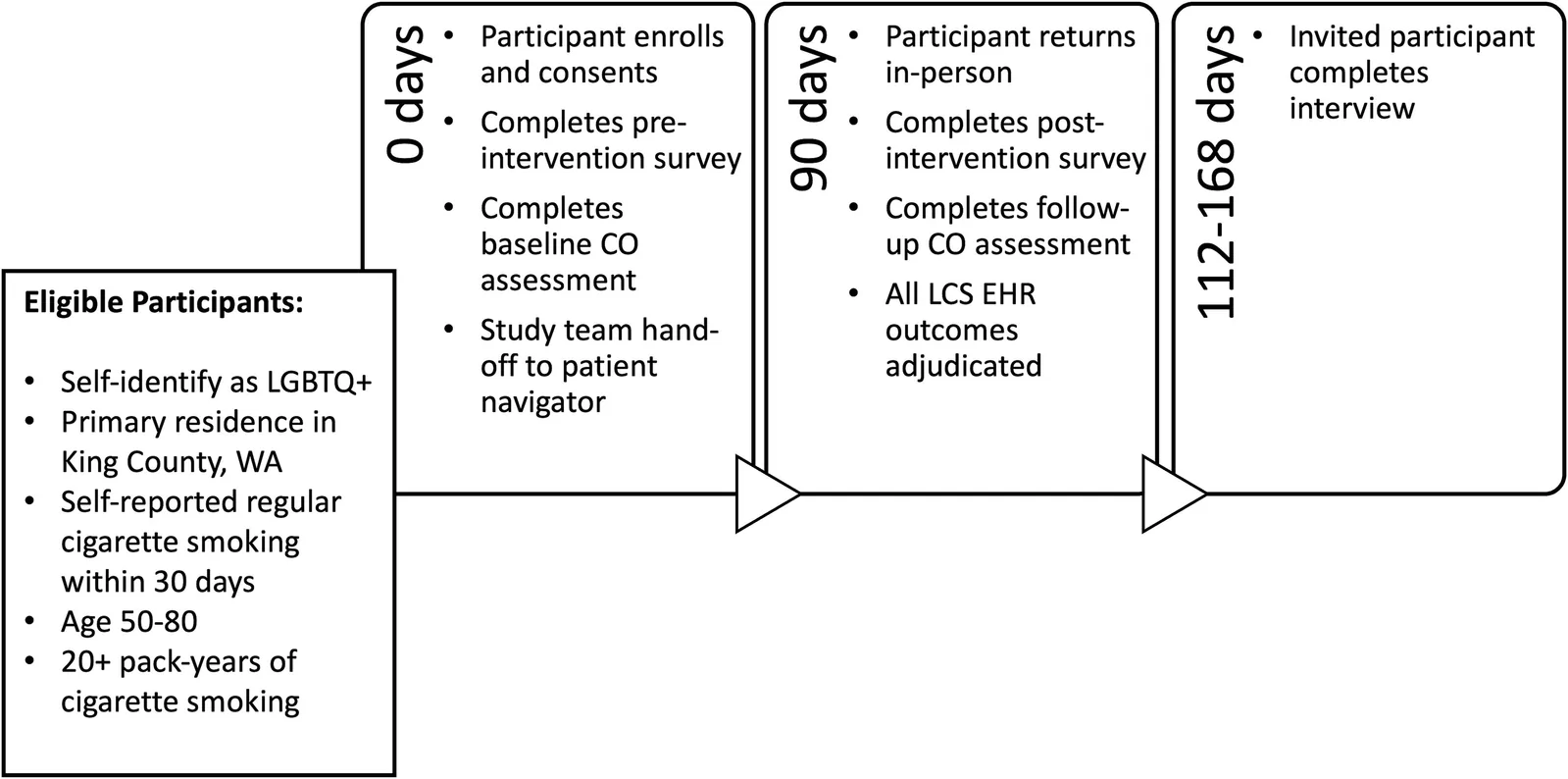
Participant eligibility and flow through study data collection.

**Table 1:** Demographic data of participants in trial, including subset who completed post-intervention survey and interviews.

Characteristic	Trial participants (*n* = 41) Median (IQR) or n(%)	Post-intervention survey participants (*n* = 34) Median (IQR) or n(%)	Interview participants (*n* = 15) Median (IQR) or n(%)
**Age**	60 (57–65)	60 (57–65)	61 (57–66)
**Sex assigned at birth**			
**Male**	31 (76%)	24 (71%)	10 (67%)
**Female**	10 (24%)	10 (29%)	5 (33%)
**Gender identity**			
**Male**	27 (66%)	21 (62%)	8 (53%)
**Female**	9 (22%)	9 (26%)	4 (27%)
**Transgender**	2 (4.9%)	2 (5.9%)	1 (6.7%)
**Two-spirit**	1 (2.4%)	0	0
**“I use a different term”**	2 (“non-binary”) (4.9%)	2 (“non-binary) (5.9%)	2 (“non-binary”) (13%)
**LGBTQ+ identity** ^**1**^			
**Gay**	25 (61%)	19 (56%)	7 (47%)
**Lesbian**	6 (15%)	6 (18%)	3 (20%)
**Bisexual or Pansexual**	3 (7.3%)	3 (8.8%)	1 (6.7%)
**Transgender**	3 (7.3%)	3 (8.8%)	2 (13%)
**Gender diverse including** **non-binary, third gender,** **two-spirit or other gender**	4 (9.8%)	3 (8.8%)	2 (13%)
**Prefer not to say**	1 (2.4%)	1 (2.9%)	1 (6.7%)
**Race1**			
**American Indian/Alaska Native**	6 (15%)	5 (15%)	4 (27%)
**Black or African American**	6 (15%)	4 (12%)	2 (13%)
**White**	33 (80%)	27 (79%)	10 (67%)
**Asian**	2 (4.9%)	1 (2.9%)	0
**Other**	2 (“multi-racial”) (4.9%)	2 (“multi-racial”) (5.9%)	2 (“multi-racial”) (13%)
**Hispanic or Latinx**	2 (4.9%)	2 (5.9%)	2 (13%)
**Highest grade completed**			
**Less than high school**	1 (2.4%)	1 (2.9%)	1 (6.7%)
**High school**	9 (22%)	6 (18%)	5 (33%)
**Some college**	14 (34%)	11 (32%)	5 (33%)
**College**	13 (32%)	12 (35%)	4 (27%)
**Graduate or post-college professional school**	4 (9.8%)	4 (12%)	0
**Annual household income**			
**<$15 000**	10 (24%)	8 (24%)	2 (13%)
**$15 000–$29 999**	12 (29%)	10 (29%)	6 (40%)
**$30 000–$50 000**	2 (4.9%)	2 (5.9%)	1 (6.7%)
**>$50 000**	13 (32%)	12 (35%)	5 (33%)
**Don’t know**	3 (7.3%)	2 (5.9%)	1 (6.7%)
**Prefer not to answer**	1 (2.4%)	0	0
**Medical insurance** ^**1**^			
**Private or HMO**	13 (32%)	11 (32%)	4 (27%)
**Medicare**	19 (46%)	16 (47%)	8 (53%)
**Medicaid**	14 (34%)	11 (32%)	5 (33%)
**Pack-years of smoking**	28 (23–50)	35 (23–50)	24 (20–50)
**Reported prior quit attempts**	38 (93%)	31 (92%)	14 (93%)
**Reported** $\boldsymbol{\ge}$ **3 medical visits per year**	27 (66%)	22 (65%)	8 (53%)

^1^more than one response allowed.

**Table 2:** Summary of themes within topical domains.

Domain	Theme	Definition
Intervention Success	Flexibility in LCS navigation and smoking cessation counseling	Participants appreciated flexible and personalized communication methods with the navigator in regard to mode and frequency of communication, as well as whether they preferred to initiate contact or to be prompted with check-ins.
	Longitudinal relationships with iterative contact fits process	Smoking cessation and LCS are processes, and best addressed by a longitudinal relationship with iterative contact with the navigator.
	Navigation facilitates confidence and comfort	As opposed to fractured healthcare, patient navigation improved confidence and comfort in the LCS and smoking cessation processes by providing information, validating the difficulty of quitting smoking, and being a longitudinal accountability partner.
	Navigator supported personalized and multimodal approaches to smoking cessation	Despite chronic smoking histories and prior quit attempts, few participants had received guideline concordant smoking cessation therapy. The navigator combined counseling, app-based approaches, and access to pharmacologic tools to provide novel and personalized approaches.
	CO monitor is a novel approach to point-of-care smoking feedback	Participants appreciated the objectivity of the CO monitor as a motivator for smoking cessation and several expressed interest in having it at home as a longitudinal accountability aid.
LGBTQ+ Care	Social and environmental barriers	Participants described the significance of smoking in the LGBTQ+ community as being a barrier to cessation given its role in fostering social bonds.
	Historic discrimination impacts smoking cessation	Discrimination related to LGBTQ+ identity and smoking status resulted in barriers to seeking care such as shame, fear of failure and health consequences, and reactance towards authoritative cessation recommendations.
	Preference for non-judgmental approaches	Participants appreciated a balanced approach to smoking cessation guidance that withheld judgment and was understanding of the difficulty of quitting.
	Variable response to LGBTQ+ tailored care	While some appreciated and emphasized the importance of an LGBTQ+ tailored intervention to facilitate a safe environment, others did not find this to be necessary.
Future Adaptations	Recommendation for education of cancer and transparency on tailored approach to LGBTQ+ people	Some participants wanted more education on lung health. Given the historical discrimination of the LGBTQ+ community, participants emphasized the importance of clearly explaining the rationale for an LGBTQ+ navigation program.
	Inclusion of trusted PCPs in intervention	Participants resisted direct-to-patient recruitment and programming and preferred inclusion of PCPs in both recruitment and programming
	Discussion of financial risks	Participants worried about costs associated with screening and smoking cessation medications which acted as barriers to seeking these which could be ameliorated by the navigator.
	Interest in community connection and peer support	The most common suggestion was for the development of a smoking cessation support group to replace potential lost social bonds from smoking and provide mentorship and solidarity in the cessation process.
	Interest in more intense support	Some participants recommended more frequent contact and more barrier-focused resources to address personal challenges in smoking cessation and obtaining LCS

LCS, lung cancer screening; PCP, primary care provider.

### Implementation success

Themes related to implementation success included aspects that were considered acceptable and led to successful participation in navigation (Supplemental Table 1).

#### Flexibility in communication

Participants expressed different preferences regarding the mode and frequency of communication and appreciated flexibility and personalization from the navigator. One noted, “options are wonderful because not everyone’s going to do the same thing,” and another, “the only thing I can say is let the patient have their choices.” Some preferred the convenience of electronic communication via email or patient portals with participants noting, “I utilize MyChart a lot,” and, “it was extremely effective, the email.” Others preferred and appreciated paper records as many did note challenges with technology as a barrier: “I never remember my passwords, so I don’t use it.” Similarly, in pre-intervention surveys, participants had differing preferences for health information being web-based, written or delivered through conversations with providers.

#### Longitudinal and iterative contact

Participants appreciated the navigator’s longitudinal availability and assistance. For smoking cessation, the navigator adapted cessation counseling to meet participants’ dynamic needs and competing life stressors. One participant highlighted the importance of having someone with them on their smoking cessation journey: “to me, it’s just somebody’s listening to you talk, then at least you have a witness.” For LCS, the navigator guided participants through points of confusion. For example, one participant said, “they helped me with scheduling and directions, and they made it really easy to go there and … find the testing area.” Later, the navigator was able to provide clarity regarding unfiltered patient portal results: “on the MyChart, the way the screen stuff came through, I didn’t understand it, so I was able to ask. And when I want to ask my doctor … it takes like six to eight weeks for me to get in.”

#### Navigation facilitates confidence and comfort

For many, the navigator enhanced confidence and comfort to pursue LCS and smoking cessation. The navigator provided reassurance and anticipatory guidance: “the questions that I had, he answered right there, and so it made it really easy and I knew how to proceed”, and, “you know, ‘hey, this is what’s going to happen,’ you know? And I really appreciated that.” The navigator also helped those experiencing anxiety feel more comfortable with LCS: “[it] was hard that I was having anxiety … [they] were exceptionally supportive of the screening process and just really happy to talk about it.” Surveys provided evidence that increasing comfort may be needed in the LCS setting, as 24% of participants reported “worry” about lung cancer either “Sometimes” or “Fairly Often.”

#### Navigator supported personalized, tailored and multimodal approaches to smoking cessation

The navigator provided guideline-concordant therapy with flexible and personalized approaches. Most had tried quitting with various approaches, but very few had received guideline–concordant smoking cessation therapy combining medication and counseling, for example, “I never had worked with a counselor. I had used nicotine gum before, but I had never used a patch.” Many appreciated specific strategies from counseling, for example, the importance of having “something to do with my hands in social situations or sorts of things so you know with when I have morning coffee with friends or something out back, you know that’s a trigger.” Some found app–based smoking cessation aids helpful: “I thought the tools he offered me were really great … one time, he offered, like, an app and for some reason I couldn’t get into it and we kind of troubleshooted that …”.

#### Carbon monoxide monitor may be a useful patient–facing tool

A carbon monoxide (CO) monitor was used at study enrollment and day 90 visit largely to confirm biochemical abstinence, but participants expressed interest in the CO monitor as a longitudinal tool to provide feedback on their smoking cessation progress. Some described it as an accountability partner, for example, “if it was something where I was getting it checked more often, and I had to do it at home and then keep a journal of it or whatever, then it would significantly increase the chances of me … quitting smoking.” Others were motivated by seeing the number go down: “let’s say you have ten sessions with somebody over ten weeks, right? You blow and one day it’s an eight, and then the next time it’s a six, and then, you know, you’ve quit and you’re so excited to go to get that blow to be a zero.” Conversely, some participants experienced stigma or anxiety using the CO monitor in front of providers, saying, “it’s kind of a trigger for the shame of having done the dumb thing in the first place.”

### LGBTQ+ care

Themes under the domain of LGBTQ+ Care characterized the impact of historical discrimination and specific social dynamics. They also explore the benefits and challenges in tailoring an intervention to a marginalized group (Supplemental Table 2).

#### Social and environmental barriers to LCS & smoking cessation

Many participants identified high rates of smoking in the LGBTQ+ community as a social barrier to smoking cessation attempts. Some related this to “bar culture,” saying, “I think that’s from years of being in the bars and that kind of stuff.” Several participants highlighted smoking as part of their experience being in the LGBTQ+ community. For example, one described how “a lot of the smoking in our community is about community.” Some grounded this in the history of communal trauma experienced by this population and identified smoking as a stress reliever: “smoking in our community is very much about connection, and it’s also, you know, in general, it’s a way to manage your emotions a lot of times anxiety.” Another explained how they would need to navigate social barriers in their cessation plan: “you’re going to be with friends, you’re not going to be in a situation you’ve set up to [not smoke].”

#### Historic discrimination impacts reactions to smoking cessation messaging

Participants described a complex interplay of historical LGBTQ+ discrimination, shame related to smoking, and negative reactions to some cessation messaging that intersected as barriers. Less than half (46%) reported always being respected by healthcare providers in the last year. One participant described the intersectionality of discrimination based on smoking and being LGBTQ+, saying, “being a part of the community, for me personally, and the connection with me smoking it just was … like feeling discriminated [against], you know … in the medical community.” Some specifically described how this impacted interactions with healthcare providers: “it’s kind of like, ‘oh, here we go these losers, these smokers, they are just killing themselves’ … I’ve got a relatively thick skin but you always kind of have the reserve that the degree of care that you’re going to get is based, you know, upon how you’re probably morally being judged.”

#### Preference for non-judgmental approach to smoking cessation

Participants approved of the non-judgmental approach to smoking cessation counseling offered in this intervention. Specifically, participants noted the importance of balance: “when it’s really pushy I don’t respond, I don’t respond, I just ignore it, but a little bit of push is good,” and “I’m someone who doesn’t want a heavy hand and I think that working with this program, I didn’t ever get a heavy hand … if it hadn’t been that way, you know, then I probably wouldn’t have continued.” Some described how a patient-centered approach to cessation attempts helped build confidence in their ability to reduce consumption. As a participant said, “I’m smoking 75% less right now than what I was when we started this. I’m happy. I’m going to finish beating this.” Another appreciated that “you guys let me quit it on my terms.”

#### Variable responses to LGBTQ+ tailored care

While most participants favored an LGBTQ+-tailored intervention and highlighted the importance of representation and safe spaces, others expressed that they did not feel this was necessary. One noted the benefit that “I didn’t have to hide my identity, you knew who I was,” with another emphasizing safety: “I was really drawn to it as I solicited the service because it was LGBTQ, and I thought that felt really safe to me.” Another highlighted the particular benefit of comfort in the context of smoking cessation, saying, “you’re always going to be more comfortable where you feel like you’re being accepted and you’re safe, and when you’re dealing with something like, say gender identity on top of trying to do something like change of behavior that’s an addiction.” Conversely, some did not find any particular value to LGBTQ+ tailored care, sharing that “yeah, no, for me, no, I know some people who that would be their preference, not me,” and “to me that’s not important, but it might be to others.”

### Future adjustments

Themes regarding future adjustments highlighted more education as well as transparency regarding tailoring to the LGBTQ+ community, connection with primary care clinicians, upfront financial guidance, incorporating peer support, and selective need for additional barrier–focused resources (Supplemental Table 3).

#### Education and transparency in tailored approach

Many participants commented on residual questions and education needs regarding lung cancer and LCS after completing the navigation intervention. Also, some encouraged increased clarity regarding the rationale for LGBTQ+-tailored care. One explained that “making that clear is important so that nobody takes the wrong idea … starts thinking, what am I, a guinea pig?” To assuage these concerns, some recommended that investigators explain that the LGBTQ+ community was identified as a group with higher rates of smoking that might benefit from a tailored approach to care. For example, “[make it] perfectly clear that it’s based on because there’s a higher rate of people that are being harmed in this area.” Many also wanted to make sure tailoring did not involve unsolicited review of sexual orientation and gender identity data in the electronic health record. As a few participants said, “specifically targeting individuals … makes me cringe so hard,” and “I wouldn’t like it at all … there’s too many people [messing] around with my privacy.”

#### Inclusion of primary care clinicians

Some participants wanted their PCPs to be more connected to the process including involvement in shared decision making, LCS results delivery, and recruitment. Regarding using PCP’s to support connection to the program, one participant stated, “it’s totally fine for it to be brought up as an option at a Wellness visit … knowing that these services are available would be great.” Another explained how this was better in regard to privacy concerns, “I mean, you’re not asking the PCP to share it with you, you’re just asking them to share it with their patients, so that to me, in terms of privacy, that’s totally fine.”

#### Discussion of financial risks

Given the complexity of medical billing and insurance coverage, participants expressed fear of financial barriers to care. In surveys, several reported delaying care in the past year because they couldn’t afford co-pays (12%), the deductible was too high (10%) or they had to pay out of pocket (10%). One described high cost of smoking cessation medication: “Nicorette, the smoking, the help stop smoking aids are not cheap, so that was a deterrent and drawback with me with this stuff, too.” Others described uncertainty with screening costs and insurance coverage: “I’m sure it costs a lot of money, and if I would, this is, you know something that I would be billed for.” Another explained the importance of upfront financial guidance, “the biggest part of it is people knowing that it’s available to them out there, and that’s a lot of what’s covered, what isn’t, what’s out of pocket costs.” Many participants felt this could be relieved by personalized counseling regarding insurance coverage.

#### Interest in community connection and peer support

Many participants recommended support groups to ameliorate the isolation they experienced and provide peer support. One participant described how this might help replace the community they had with smoking: “I think it’s really important to find other ways to foster that community, sense of community … it does feel very isolating to give up this. This way that you had previously connected with other people.” Another described, “the only thing I could think of would be to foster some sort of community. I felt pretty isolated through the process, my check-ins with navigator were very much like life raft.” Some stated a preference for such a group to be specific to the LGBTQ+ community, recommending, “a little bit more of an LGBT support group around quitting smoking.”

#### Desire for more intense support when needed

Some participants recommended additional contact from the navigator which may have assisted with care completion. For example, one participant said, “If he had reached out, let’s say, once a week, I probably would have relented and reached back out to him.” Others reported that they needed more specific barrier directed support, and transportation was mentioned specifically by a few participants with one participant reporting, “Was not able to complete lung cancer screening … Needed more support to get to and from locations.” Seventeen percent of patients reported transportation had significantly delayed their medical care in the last 12 months.

## Discussion

LGBTQ+ participants who were currently smoking and eligible for LCS were enrolled in a 90-day pilot study of a patient navigation intervention to improve LCS care completion and assist with smoking cessation. In primary effectiveness outcomes, guideline concordant LCS did increase during the study and many participants had at least short-term cessation, though knowledge of LCS did not improve in pre-post comparisons (LKS 5.0 [SD1.2] vs. 5.1, *P* = .91; LCS-12 5.5 [SD 2.0] vs. 6.0 [SD2.1], *P* = .21).^20^ In this manuscript we integrated multi-method participant data to reveal determinants of implementation success, themes related to LGBTQ+ care and suggestions for future adaptation.

Several aspects of the intervention highlighted in themes may have driven acceptability of the program. In particular, a longitudinal navigation approach which encouraged a supportive 1:1 relationship between a provider and a patient and differs from usual medical care models.^30^^–^^32^ Additional aspects of navigation like flexibility, long-term relationships, personalization, and multi-modal approaches make it an excellent model to provide lung cancer prevention care. Much of the navigation care paradigm grew from supporting patients through complex cancer care continuums and LCS navigation interventions have demonstrated both efficacy and patient satisfaction in a number of underserved settings.^33–36^ This is the first navigation intervention we are aware of to place equal emphasis on smoking cessation and LCS care, and while effectiveness data is short-term in a small sample, it demonstrates that navigation may be well-suited to support both behavior change and care completion activities.

As an intervention developed and tailored to LCS-eligible LGBTQ+ individuals, themes related to LGBTQ+ care mirror evaluations of prior LGBTQ+-focused smoking cessation interventions. Culturally tailored smoking cessation programs for LGBTQ+ people have been demonstrated to be acceptable and effective.^37–39^ LGBTQ+ people may be particularly receptive to interventions that emphasize non-judgmental and patient-centered approaches to smoking cessation given the community’s complex history with stress and discrimination both inside of and outside of medical environments.^40^^,^^41^ In this context, participants emphasized how personalized CO monitors provide a private biofeedback option that enhances self-efficacy and motivation for smoking cessation. Social support may also be a particularly essential component of effective LGBTQ+ focused interventions.^42^ In our study, while most participants appreciated LGBTQ+ tailored care, some highlighted that tailored care can raise suspicions for ulterior motives or unequal care, and that it is important to explain the rationale for tailored care and emphasize patient privacy around disclosure and identity.

There were novel aspects of certain themes that merit further investigation as methods to enhance lung cancer prevention in this community, and perhaps in others. Primarily, combining smoking cessation counseling with LCS navigation is a promising intervention to provide both primary and secondary prevention of lung cancer. Smoking cessation counselors may be particularly effective as LCS navigators for individuals who are currently smoking. Also, there was support for combining peer support with navigation. This may reflect the social isolation many older LGBTQ+ adults reported and provide an additional mode of supportive care to enhance both cessation and LCS care completion.^43^ We are not aware of evidence-based interventions that combine navigation and peer support in the lung cancer prevention setting. Future iterations should also consider ensuring the primary care clinician is involved and integrated in the navigation process and consider the intensity of the intervention given substantial resource requirements which may limit its accessibility.

Strengths of the study include the novelty of the intervention and the integration of broad data in the evaluation including participant data from qualitative interviews with both free–text survey responses and close-ended responses. High participant completion of post-intervention surveys and a broadly representative sample for interviews enhanced triangulation of data to support summary themes. Limitations of this study include its small sample size, its restriction to a single geographic area, and over-representation of participants who identified as gay men. Purposive sampling of interview participants, while encouraging diverse viewpoints, may have led to a selection bias towards acceptability in the interview sample. Given the small sample size, and the diversity within the LGBTQ+ community and the importance that intersectional health–related social needs likely have on navigation outcomes, it will be important for external validity to evaluate this intervention in other settings and included gender-diverse and racial and ethnic minority LGBTQ+ populations. The lack of comparison group also greatly limits attributing LCS completion and smoking cessation directly to the intervention and further evaluation should, ideally, include a randomized trial. Future study should also assess sustained outcomes including long–term smoking cessation and ongoing adherence to annual LCS.

### Implications

This summative evaluation of a combined smoking cessation and LCS care navigation intervention for LGBTQ+ adults demonstrated that participant success was likely related to features of patient navigation care including relationship building and flexibility and enhanced by a non-judgmental and LGBTQ+-tailored approach. Further adaptations to improve success could include more lung cancer and LGBTQ+-tailored education, overt and personalized discussions of financial risk and using navigation in combination with a peer support model. This model could represent an effective approach to combined LCS and smoking cessation care in other minoritized populations.

## Supplementary Material

Supplemental_Tables_FINAL_ntag136

## Data Availability

De-identified qualitative data and summarized datasets created during the current study will be made available from the corresponding author on reasonable request.
