# Supplementary material for: A mixed-methods evaluation of a patient navigation intervention to support lung cancer prevention in LGBTQ+ adults
Source: Nicotine Tob Res. 2026 Jun 19;28(11):1951–60. doi: 10.1093/ntr/ntag136 (PMC13687886; doi:10.1093/ntr/ntag136)
Supplement: Supplementary file 1 [file supplemental_tables_final_ntag136.docx]

**Supplemental Tables**

**Supplemental Table 1. Themes mapped to the “Intervention Success” domain with definitions and exemplary quotes.**

| Theme | Definition | Interview Quotes | Pre- or Post-Survey quantitative data | Survey responses |
| --- | --- | --- | --- | --- |
| *Flexibility in LCS navigation and smoking cessation counseling* | Participants appreciated flexible and personalized communication methods with the navigator in regard to mode and frequency of communication, as well as whether they preferred to initiate contact or to be prompted with check-ins. | -“If it's just a text or something, it can be taken out of context because maybe they're reading it and it not a way that it was actually said, you know what I'm saying? So yeah, there definitely has to be that human contact, at least where you can hear somebody when it comes to something very personal”  -“I think some people probably would prefer it to just come through MyChart. I personally think it should come from a person so they can kind of answer some questions and if you I think if you have an interaction with another human being, it's going to stick more than just something. Because if you if a primary talks to you about it, you're going to have to come up with excuses for not doing it. Whereas if you read it, you know what I mean. Like if you read it, you can just brush it off and nobody's there to hold you accountable for brushing it off, so yeah”  -“The way that he had it set up really worked out well for me because it wasn't like bugging me every week or every whatever. It was like, okay, we'll do one now, and then in a couple of weeks we'll do another one. So it was really spaced out well”  -“Once a week, or every other week, every ten days, get together for half hour, an hour session, even if it's over a cup of coffee or over the telephone, just to kind of reaffirm everything and make sure that I'm keeping me in line” | How do you like to receive health information?^1^:   - Web-based: 25 (61%) - Video-based: 0 - Written materials: 10 (24%) - Talking directly with doctor: 30 (73%) - Talking directly with other healthcare providers: 23 (56%)   “My navigator is easy for me to reach”^2^:   - Agree or Complete Agree: 30 (88%) | “Sufficient info around options-made it easy to understand. Phone option vs. in-person was helpful…Flexible scheduling.”  “Flexible phone/email communication.”  “Ease of use of mychart and phone.”  “Regular check ins, accountability. Phone calls appreciated.” |
| *Longitudinal relationships with iterative contact fits process* | Smoking cessation and LCS are processes, and best addressed by a longitudinal relationship with iterative contact with the navigator. | -“It's a habit that I have, that I'm just, I can't say willing because I'm willing to stop, but I just don't have it in me to stop right now”  -“He was always there to answer any of my questions and provide avenues to go forward”  -“He gave me different options for transportation, but for me the bus was the quickest”  -“One of my favorite things about the process was just like I had received my results on MyChart and but then Navigator also checked in”  -“I had already looked at my results and kind of knew what they meant, but, yeah, he did, he was able to actually clarify a few things too. So, it was, I didn't understand all of it, and yeah, it was great”  -“There was something I either wasn't understanding or had wrong or something and yeah, he, I remember, he clarified that and it was just, yeah, it was really helpful. And he did it pretty, it was timely, after my results came in, he just got in touch with me pretty quickly so, yeah, it was good” | “My navigator cares about me personally”^2^:   - Agree or Complete Agree: 26 (76%) | “Encouraged to make an effort through discussion with [navigator], was easy to communicate with, appropriate amount of communication, ongoing services is a plus.”  “Direction to resources, repeated check-ins (especially with cancer screening.”  “Good listener—recalled personal stories and referenced later.” |
| *Navigation facilitates confidence and comfort* | As opposed to fractured healthcare, patient navigation improved confidence and comfort in the LCS and smoking cessation processes by providing information, validating the difficulty of quitting smoking, and being a longitudinal accountability partner. | -“It's very hard to get a human being on the phone anymore without going through twenty different robots”  -“I have a pretty good grasp on this stuff now. Like I said, I'm determined. I'm going to quit”  -“And that helps person in my position, being a smoker and wanting to quit, you have the knowledge that I'm trying to get. Actually, I have the knowledge, I just have to put it to use”  -“They dumbed it down to my level so I could understand them and we could have a regular conversation. So, I think I went into it open minded, and I came out of it with the knowledge that I felt safe going through with it and understanding the results and then following it up with the surgery and having them remove the cancer”  -“There's more to this than I actually even imagined. You know, there are resources and there is the way if you really want to get screened”  -“I thought the screening process was amazing. I thought it was fast”  -“He made me feel 100% comfortable” | Do you worry about getting lung cancer?^1^:   - Never: 7 (17%) - Rarely: 24 (59%) - Sometimes: 9 (22%) - Fairly often: 1 (2.4%)   How often did your navigator tell or give you information about your health and health care that was easy to understand?^2^:   - Most of the time or Always: 33 (97%) | “Calmy and courteously accepted opinions, supported efforts.”  “In my interaction with [the navigator] he was attentive, kind, and informative.”  “Information around lung cancer provided helpful, put at ease by results.” |
| *Navigator supported personalized and multimodal approaches to smoking cessation* | Despite chronic smoking histories and prior quit attempts, few participants had received guideline concordant smoking cessation therapy. The navigator combined counseling, app-based approaches, and access to pharmacologic tools to provide novel and personalized approaches | -“I needed a different approach, and I just didn't know how to do it”  -“Having someone there to bounce ideas off of or give you ideas of things for changing your behaviors so you can quit, that was nice to have”  -“What I liked about talking with [navigator] about quitting smoking was his constant reassurance and providing me with tools that if there was a trigger that triggered something, what I could do and how to maneuver through that”  -“If it's, you know, 7:30 and typically you're going outside for a smoke, maybe you should go upstairs and fold laundry or listen to music or do something different. So, he was super helpful in that and just supportive”  -“I really loved his approach. He had a very multi-tiered approach, any issue I would bring up he would have resources, he very creative, just lots and lots of ideas” | Since starting the study, which of the following did you use to help quit smoking?^2^:   - Doctor or provider counseling: 0 (0%) - Navigator counseling: 10 (29%) - Telephone Quit Line: 1 (2.9%) - Friend or Family assistance: 4 (12%) - Nicotine replacement therapy: 14 (42%) - Prescription medications: 3 (8.8%) - Other: 3 (8.8%) | “Understood unique needs (different for each person) as quitting smoking.”  “Not ready to quit but tools have been offered (and appreciated). Not pushy on quitting. Options offered, including his contact and continued resources…”  “free NRT and provided ample options, very positive experience.” |
| *CO monitor is a novel approach to point-of-care smoking feedback* | Participants appreciated the objectivity of the CO monitor as a motivator for smoking cessation and several expressed interest in having it at home as a longitudinal accountability aid. | -“That little breathing machine thing that I did that I blew off the chart. I want one of those to see so I can monitor my own health”  -“You blew it off the charts, that doesn't really happen. And it's like, great. And then in the end, when we did it again and it was only halfway through, I only went halfway up the chart this time, the measured chart, so that made a really big, good impact on me for that. Okay, I can do this. And it's made that much of a difference in that amount of time”  -“It's something that would make a person accountable without being intrusive without it feeling like a punishment, or where you're being monitored”  -“I thought it was anxiety provoking… it felt kind of vulnerable to do it like in front of you”  -“I think it would make me feel proud to be able to, like, come in with numbers where you guys could see”  -“You'd have to be accountable without it being intrusive. Like where it was almost like you get a bad mark if you know or whatever. Because in one respect you do need some accountability if you know you're doing something that's detrimental to your life if you don't quit. But in the same respect, like I was saying with any kind of addiction scenario, people generally don't want to be told what they have to do or anything” |  |  |

LCS=lung cancer screening; CO=carbon monoxide; 1 Pre-survey question; 2 Post-survey question

**Supplemental Table 2. Themes within the domain of “LGBTQ+ Care” with definitions and exemplary quotes.**

| Theme | Definition | Interview Quotes | Survey quantitative data | Survey responses |
| --- | --- | --- | --- | --- |
| *Social and environmental barriers* | Participants described the significance of smoking in the LGBTQ+ community as being a barrier to cessation given its role in fostering social bonds. | -“What’s really important for you guys to realize is that there is so much trauma involved in being queer in this in this community, you've got to assume that people have experienced trauma related to it, not just stress... It might be helpful for you guys to find out and maybe you did ask me this, but when people started, or what you know, started using smoking or tobacco as a way you know when they started really using it and why.”  -“Plus my husband smokes so it's, you know, it doesn't help when one person, one is smoke and the other one he doesn't, you know what I mean?”  -“I never smoked because I felt like I had to. It was just like a social thing.”  -“I want a cigarette or I want to hit a vape when I'm around those people”  -“That community tends to be, you know, bar driven.” |  |  |
| *Historic discrimination impacts smoking cessation* | Discrimination related to LGBTQ+ identity and smoking status resulted in barriers to seeking care such as shame, fear of failure and health consequences, and reactance towards authoritative cessation recommendations. | -“People generally don't want to be told what they have to do or anything”  -“I have kind of been in the closet around my trying to quit smoking, if you will. When I first tried to quit a while back, I didn't even tell anybody because I don't want people to then check in with me, you know what I mean? Although that's a very nice thing, people want to be helpful, I do understand that, but it wouldn't have felt helpful to me. It would have triggered my shame stuff.”  -“It really scares me because I've done a number. I've been smoking for years and years and years”  -“There's the lifestyle that I've led, and you know, I know I've been lucky in a lot of ways and I'm really kind of scared to find out. But it's something that I know I need to have done because it's better to know early than to find out later.”  -“Finding the result out alone, that is, I think, why probably a lot of people don't do it. They just don't want to know if there is something.” | In the past year, how often have you either delayed or not gone to see doctors of healthcare providers because they were different from you”^1^:   - Most of the time 2 (5%) - Some of the time 10 (25%)   In the past year, how often were you treated with respect by your doctors and healthcare providers?^1^:   - Always: 19 (46%)   “I did not face discrimination during smoking cessation counseling”^2^:   - Agree or Strongly Agree: 31 (89%) |  |
| *Preference for non-judgmental approaches* | Participants appreciated a balanced approach to smoking cessation guidance that withheld judgment and was understanding of the difficulty of quitting. | -“He wasn't, like, pushy about it. I felt better because I said I hadn't done anything and I’ve got nothing to report to you but he was like, ‘you know, that's fine’.”  -“No, there wasn't anything bad like that, or derogatory or anything. No brow beating or putting me down, making me feel less of a person or anything like that. He was very cordial.”  -“I mean it just it's that was like my biggest fear going in both times is like, oh, god, I’ve got nothing to say because I haven't done anything”  -“I didn’t feel like I was being, you know, judged. He shared some of his personal experiences with smoking, which I though was insightful.” | “I felt respected by the navigator”^2^:   - Agree or Strongly Agree: 31 (89%)   “I felt heard by the navigator”^2^:   - Agree or Strongly Agree: 31 (89%) | “Not pushy on quitting.”  “Shared personal experience.” |
| *Variable response to LGBTQ+ tailored care* | While some appreciated and emphasized the importance of an LGBTQ+ tailored intervention to facilitate a safe environment, others did not find this to be necessary. | -“There's an immediate feeling of trust, of camaraderie because you know that at the very least, this other person understands that.”  -“ I was encouraged that there was some kind of study that specifically wanted to reach out to the LGBTQ plus whatever, because historically so much of our community centered around meeting in places where smoking and vaping is kind of encouraged.”  -“Somebody my age and stuff probably not so much. But I think that that's definitely a factor for the younger generation” | “It is important that the lung cancer screening process is tailored to me and my community.”^1^:   - Somewhat or Strongly Disagree 6 (18%) - Somewhat or Strongly Agree: 28 (82%) | “Targeted LGBTQ specifically and knowledge that smoking and identity were connected." |

1 Pre-survey question; 2 Post-survey question

**Supplemental Table 3. Themes within the domain of “Future Adaptations” with definitions and exemplary quotes.**

| Theme | Definition | Interview Quotes | Survey quantitative data | Survey responses |
| --- | --- | --- | --- | --- |
| *Recommendation for education of cancer and transparency on tailored approach to LGBTQ+ people* | Some participants wanted more education on lung health. Given the historical discrimination of the LGBTQ+ community, participants emphasized the importance of clearly explaining the rationale for an LGBTQ+ navigation program. | -“Because anytime where, especially being a person of color too, anytime where you're coming into a situation where there's a specific group of people being isolated for anything, the first thing you want to know is why.”  -“Why is the program specifically designed for us? As a specific community, is there a reason for that? Because that might leave a bad taste” | Has a doctor talked to you about lung cancer screening before?^1^:   - No/Not sure: 18 (43%)   “After participation, I still have questions about lung cancer screening”^2^:   - 14 (41%) Somewhat or Strongly Agree | “Not sufficient description of screening and it’s benefit.”  “Needed…more intentional messaging, materials, etc.” |
| *Inclusion of trusted PCPs in intervention* | Participants resisted direct-to-patient recruitment and programming and preferred inclusion of PCPs in both recruitment and programming | -“I was thinking maybe reaching out to all the different docs… that they know about, you know the program.”  -“If you're asking [PCPs] to share it with their patients, then I think I don't see any problem with that at all, I see that as a really good idea.”  -“If my doctor had recommended it or mentioned it to me ahead of time that he was going to refer me to that, that would be great.” |  | “Not able to directly communicate with care team at [other health system].” |
| *Discussion of financial risks* | Participants worried about costs associated with screening and smoking cessation medications which acted as barriers to seeking these which could be ameliorated by the navigator. | -“I wasn't aware that (NRT) was available, and I just thought that was something, I'm used to being told that those are things that are cost out of your own pocket. But yeah, because that stuff, even in Walmart, it's kind of spendy you know.  -“It's expensive to buy it over the counter and I think if you get it through, I don't know, like your health insurance or something, you only get like a month  -“It's probably would probably, I would imagine it would be if not up in the thousands at least, like you know a few hundred or whatever. With this kind of screening.”  -“ I would like to do it. And I just got my Medicare approved, so I don't even have a provider yet, I know, you know now it's like I don't know what to do next” | Reported financial concerns that delayed care over past year^1^:   - Couldn’t afford co-pay: 5 (12%) - Deductible too high: 4 (9.8%) - Had to pay out of pocket: 4 (9.8%) | “Limitations of navigator in billing issues.” |
| *Interest in community connection and peer support* | The most common suggestion was for the development of a smoking cessation support group to replace potential lost social bonds from smoking and provide mentorship and solidarity in the cessation process. | -“Community is important, right? I think it's really important. So that would be something that would be a good option.”  -“If there's a group or a place where as a smoker who is trying to quit and not always successful, where they can go and say, yeah, I messed up again, and have the person who is hearing those witnessing that, understand what that meant, like somebody who tried to quit smoking and couldn't. So more like a support group kind of idea.”  -“Maybe you could have little support group meetings someplace, you know, around the area that folks could sit down and talk to each other. If nothing else, just communicate and not smoke together.”  -“Sometimes I think like a group thing like AA but for smoker.”  -“Kind of like a place where you could go to, you know, like attend a group. Or, you know, where other people are going through it or what have you or get a coach.” |  | “Lack of peer support.”  “Potentially larger community recruitment methods (need exists).” |
| *Interest in more intense support* | Some participants recommended more frequent contact and more barrier-focused resources to address personal challenges in smoking cessation and obtaining LCS | “If he had reached out, let's say, once a week, I probably would have relented and reached out back to him.” | Reported barriers that delayed medical care in the last 12 months^1^:   - Transportation: 7 (17%) - Rural residence: 1 (2.4%) - Couldn’t get time off work: 2 (4.9%) - Couldn’t get childcare: 0 - Providing care to other: 3 (7.3%) - Other reasons: 13 (32%) | “Never got to the screening, required additional support.”  “Would’ve liked more communication and follow-up by phone.”  “Was not able to complete lung cancer screening…Needed more support to get to and from locations.” |

LCS=lung cancer screening; PCP=primary care provider; 1 Pre-survey question; 2 Post-survey question
